# The Evolution of Care of Cancers of the Head and Neck Region: State of the Science in 2020

**DOI:** 10.3390/cancers12061543

**Published:** 2020-06-11

**Authors:** Flora Yan, Hannah M. Knochelmann, Patrick F. Morgan, John M. Kaczmar, David M. Neskey, Evan M. Graboyes, Shaun A. Nguyen, Besim Ogretmen, Anand K. Sharma, Terry A. Day

**Affiliations:** 1 Head and Neck Tumor Center, Hollings Cancer Center, Department of Otolaryngology—Head and Neck Surgery, Medical University of South Carolina, Charleston, SC 29425, USA; yanf@musc.edu (F.Y.); morgap@musc.edu (P.F.M.); neskey@musc.edu (D.M.N.); graboyes@musc.edu (E.M.G.); nguyensh@musc.edu (S.A.N.); 2 Department of Microbiology and Immunology, Medical University of South Carolina, Charleston, SC 29425, USA; knochelm@musc.edu; 3 Head and Neck Tumor Center, Hollings Cancer Center, Department of Hematology/Oncology, Medical University of South Carolina, Charleston, SC 29425, USA; kaczmar@musc.edu; 4 Department of Biochemistry and Molecular Biology, Medical University of South Carolina, Charleston, SC 29425, USA; ogretmen@musc.edu; 5 Head and Neck Tumor Center, Hollings Cancer Center, Department of Radiation Oncology, Medical University of South Carolina, Charleston, SC 29425, USA; sharmaak@musc.edu

**Keywords:** head and neck cancer, head and neck squamous cell carcinoma, thyroid cancer, salivary gland cancer, skin cancer, COVID-19

## Abstract

Cancers that arise in the head and neck region are comprised of a heterogeneous group of malignancies that include carcinogen- and human papillomavirus (HPV)-driven mucosal squamous cell carcinoma as well as skin cancers such as cutaneous squamous cell carcinoma, basal cell carcinoma, melanoma, and Merkel cell carcinoma. These malignancies develop in critical areas for eating, talking, and breathing and are associated with substantial morbidity and mortality despite advances in treatment. Understanding of advances in the management of these various cancers is important for all multidisciplinary providers who care for patients across the cancer care continuum. Additionally, the recent Coronavirus Disease 2019 (COVID-19) pandemic has necessitated adaptations to head and neck cancer care to accommodate the mitigation of COVID-19 risk and ensure timely treatment. This review explores advances in diagnostic criteria, prognostic factors, and management for subsites including head and neck squamous cell carcinoma and the various forms of skin cancer (basal cell carcinoma, cutaneous squamous cell carcinoma, Merkel cell carcinoma, and melanoma). Then, this review summarizes emerging developments in immunotherapy, radiation therapy, cancer survivorship, and the delivery of care during the COVID-19 era.

## 1. Introduction

Cancers arising in the head and neck region are a heterogenous group of malignancies that include carcinogen- and human papillomavirus (HPV)-driven mucosal squamous cell carcinoma as well as skin cancers such as cutaneous squamous cell carcinoma, basal cell carcinoma, melanoma, and Merkel cell carcinoma. These malignancies develop in critical areas for eating, talking, and breathing and are associated with substantial morbidity and mortality despite advances in treatment [1,2]. Understanding of advances in head and neck cancer (HNC) management is important for all multidisciplinary providers who care for these patients across the cancer care continuum. Additionally, the recent Coronavirus Disease 2019 (COVID-19) pandemic has necessitated adaptations to HNC care to accommodate mitigation of COVID-19 risk and ensure timely treatment [3]. This review explores advances in diagnosis, prognostication, and management by subsite. Then, this review summarizes emerging developments in immunotherapy, radiation therapy, cancer survivorship, and delivery of care during the COVID-19 era. 

## 2. Head and Neck Squamous Cell Carcinoma 

### 2.1. Epidemiology, Prevention, and Surveillance

Head and neck squamous cell carcinoma (HNSCC) traditionally includes mucosal squamous cell carcinomas of the oral cavity, pharynx, and larynx and represents over 65,000 new cancer cases and 14,500 cancer-related deaths in the United States annually [4]. Cancers of the oral cavity, larynx, hypopharynx, and a small percentage of oropharynx are associated with smoking and alcohol consumption and have been decreasing in incidence in recent years [5]. In contrast, the primary etiologic factor for oropharyngeal SCC (OPSCC) is now HPV. Of note, cancers of the nasopharynx, paranasal sinuses, and nasal cavity may have a distinct pathogenesis from the aforementioned subsites and are discussed elsewhere [6,7,8,9]. The incidence of HPV-positive OPSCC has been exponentially rising, with an annual increase of 3.8% in Caucasian men between 1992 and 2015 [5,10]. The incidence of HPV-positive OPSCC is now 2.5 times that of HPV-negative OPSCC and is now more common than HPV-driven cervical cancer [11]. Traditionally, HPV-positive OPSCC has affected younger Caucasian men <65 years old and is strongly related to the behaviors that expose patients to increased oral HPV infection [11]. Extrapolation of recent national trends suggest that, in the coming years, older Caucasian men will make up the majority of new cases, whereas rates may plateau in younger Caucasian men [10,12]. Recent studies have also shown rates of HPV-positive OPSCC to be increasing in women as well as non-Caucasian individuals, demonstrating the widespread impact of HPV-positive OSPCC across all populations [13,14]. 

HPV vaccination can decrease the rate of oral and oropharyngeal HPV infection and may theoretically reduce the risk of HPV-related HNSCC [15]. As of October 2018, the allowable age range for the 9-valent HPV vaccination has been expanded up to 45 years of age [16], as over 7 million new HPV infections are acquired in adults over the age of 25 [17]. Unfortunately, low vaccination rates in adult (6.9%) and adolescent (49.8%) men pose a barrier to the prevention of HPV-positive OPSCC [15,18]. Heightened awareness of these preventative measures may reduce future overall cancer burden. 

New surveillance methods for the recurrence of HPV-positive OPSCC are emerging, as recurrence may occur later than and in more atypical patterns than that of HPV-negative OPSCC [19,20]. In a multi-institutional study, Fakhry et al. [21] demonstrated that the persistent detection of HPV DNA in an HPV-detecting oral rinse following treatment of HPV-positive OPSCC was associated with increased risk of recurrence and death. Similarly, Chera et al. [22] determined that detection of plasma circulating tumor (CT) HPV DNA had a high positive predictive value in detecting the recurrence of HPV-positive OPSCC, with a median lead time of 3.9 months prior to histological biopsy-proven recurrence. These studies provide a basis for future developments in post-treatment surveillance to allow for earlier detection of recurrence and timely treatment.

### 2.2. Updates from the 8^th^ edition of the American Joint Committee on Cancer Staging System

The 8^th^ edition of the American Joint Committee on Cancer (AJCC8) classification system, published in 2017, brought about many changes in the staging of HNSCC [23]. Some of the major changes included the stratification of subsite based on HPV status, the inclusion of extranodal extension (ENE) in non-HPV related HNSCC, inclusion of depth of invasion (DOI) in oral cavity HNSCC, and changing definitions of unknown primary tumors.

With inherent differences in tumor biology and a clear prognostic advantage of HPV-associated disease, the AJCC8 classification system developed a separate staging system that differentiated between HPV-positive and HPV-negative OPSCC [23,24,25]. Specifically, clinical and pathologic nodal staging as well as overall stage criteria were made distinct. Even though these cancers often have advanced nodal disease, they have clearly demonstrated improved survival rates over that of their HPV-negative counterparts [24,25]. The creation of a separate staging system for HPV-positive OPSCC has allowed for improved hazard discrimination between stages, as previous staging by the AJCC7 did not accurately reflect prognosis in this patient population [26]. Importantly, changes in HPV-positive OPSCC staging by the AJCC8 do not translate into changes in treatment recommendations [27], which are still based on the AJCC7 stage.

Carcinomas of unknown primary (CUP), presenting with isolated or multiple nodal metastases demonstrating histologically confirmed squamous cell carcinoma in the absence of an identifiable primary tumor, are common entities and make up 5–10% of HNSCC [28]. These cases traditionally undergo rigorous diagnostic evaluation including fiberoptic laryngoscopy, imaging, fine needle aspiration of neck mass, and directed biopsies of suspicious mucosal sites of the upper aerodigestive tract [27,29]. If a primary site is still not confirmed, palatine and/or lingual tonsillectomies can be performed, given the extent and laterality of nodal disease [29]. Recent studies have demonstrated a high association of CUP with HPV-positive OPSCC, as early stage HPV-positive OPSCC tumors may be undetectable on clinical and radiographic exam, yet present with early regional lymph node metastasis. In fact, over 90% of CUP malignancies are thought to be truly of HPV-positive OPSCC origin [28]. Motz et al. [30] further underscored this finding, as the authors demonstrated cases of CUP to have a high rate of HPV positivity (91%) as well a higher prevalence in a younger, male demographic. This can also explain the increasing incidence of CUP in parallel to HPV-positive OPSCC in the present era [28]. As such, the AJCC8 allows for HPV tumor marker status of these nodal metastases to reclassify these tumors as occult HPV-positive OPSCC. Additionally, as nasopharyngeal cancers positive for Epstein–Barr virus have also presented initially as CUPs, the AJCC8 allows for a positive Epstein–Barr virus tumor marker status to reclassify CUP as Epstein–Barr virus-positive nasopharyngeal SCC [23,27]. Identification of the primary site can provide important prognostic and therapeutic implications and potentially allow for more primary- and nodal-targeted treatment [31]. Additionally, those that remain truly unknown primaries after diagnostic workup cannot receive site-specific treatment, translating into poorer outcomes. These patients may also experience higher irradiation dose to certain sites or extensive irradiation of the head and neck mucosal axis, and excessive post-radiation side effects [23].

**Figure 1:**
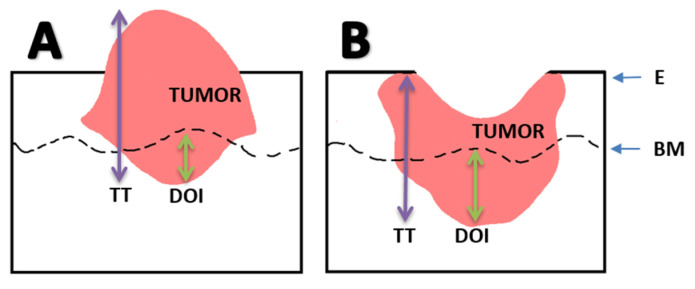
Measuring depth of invasion in an exophytic (**A**) versus ulcerative (**B**) tumor. Even though the exophytic tumor is thicker, the ulcerative tumor has a greater DOI below the normal basement membrane level. Abbreviations: Basement membrane (BM), Depth of invasion (DOI), Normal Epithelial Surface Level (E), Tumor Thickness (TT).

The new staging system also highlighted the prognostic significance of extranodal extension (ENE), which is defined as nodal disease extending beyond the lymph node capsule [23]. ENE can be stratified into microscopic or macroscopic disease, if extending ≤2 mm or >2 mm beyond the capsule, respectively [23]. ENE may upstage N-classification in HPV-negative HNSCC; however, ENE does not play a role in the nodal staging scheme for HPV-positive disease, as its prognostic role in this population remains to be elucidated. Additionally, depth of invasion (DOI), the distance measured from the epithelial basement membrane to the point of deepest invasion, is now incorporated into the T-classification of oral cavity squamous cell (OCSCC) (Table 1) [32]. Special attention must be paid to ulcerative versus exophytic tumors to accurately define the DOI (Figure 1). DOI also has an important role in determining the elective neck treatment of clinical node-negative OCSCC; DOI >3 mm has demonstrated high occult nodal metastasis rates to warrant elective node dissection [33]. Deeper invasion may signify closer proximity to local lymphovascular structures and portend a higher risk of locoregional spread. Notably, DOI is not synonymous with tumor thickness, which is defined by the diameter of the entire tumor mass from the deepest to the most superficial point [34]. Lastly, “extrinsic tongue musculature involvement” has been removed as a criterion for T4a OCSCC disease, but it is still included for both OPSCC and glottic cancers [23].

The addition of ENE and DOI to the AJCC8 staging criteria of OCSCC has been shown to improve risk stratification [35,36]. Mascitti et al. [36] demonstrated an upstaging of 23% of patients in either T- or N-classification when restaged according to AJCC8 criteria. These patients undergoing stage migration had significantly poorer disease-free survival and overall survival. When examined individually, T-classification migration based on DOI has had varying degrees of prognostic importance. However, studies examining N-classification migration have demonstrated a clear prognostic role of ENE in decreasing locoregional and overall survival. Further investigations to elucidate the optimal role of DOI in tumor, node, and metastasis TNM staging of OCSCC are warranted.

### 2.3. Treatment

Early stage HNSCC can be treated with single-modality therapy through surgical resection or primary radiation therapy (RT) [27]. Locally advanced cancers require multimodality therapy in the form of surgery and adjuvant (chemo) radiation or primary chemoradiation therapy (CRT); whereas recurrent/metastatic (R/M) disease can be salvageable with surgery, RT, and/or a wide range of systemic therapies [27]. Advances in minimally invasive surgery for HNSCC as well systemic therapy options will be discussed below. Additionally, the excellent prognosis of HPV-positive OPSCC has spurred multiple treatment de-intensification trials aiming to identify the optimal treatment paradigms to balance oncologic outcomes and treatment toxicity [37].

#### 2.3.1. Robotic Surgery

Transoral robotic surgery (TORS), a minimally invasive surgical technique, has become a common surgical modality in early stage base of tongue and tonsillar OPSCC [38]. TORS first achieved approval from the Food and Drug Administration (FDA) with the Da Vinci S, a three-armed rigid robotic system. Subsequent improvements have led to the release of the Si, Xi, and single-port (Sp) robotic systems [30,38,39]. These may better accommodate the anatomically confined regions of the head and neck.

Multiple studies have shown similar survival rates between patients treated with TORS versus RT [40,41]. Therefore, choice of curative-intent treatment requires a nuanced consideration of treatment-specific side effects and the feasibility of complete oncologic resection. In general, primary RT patients may experience mucositis, xerostomia, and soft tissue fibrosis. These are often unavoidable consequences of RT. On the other hand, post-operative complications following surgery, such as hemorrhage, infection, pain, or shoulder dysfunction from neck dissection, may result from technical error or from extensive resection to achieve oncologically sound results (e.g., sacrifice of the accessory nerve during neck dissection) [42]. TORS and primary RT both impact swallowing function, as demonstrated with feeding tube dependency rates of 0–18% after RT and 0–21% after TORS [43]. Additionally, TORS may result in the removal of portions of the tongue or soft palate, resulting in dysphagia or velopharyngeal insufficiency. Final pathology following surgery can also “upstage” the cancer, necessitating adjuvant therapy.

The ORATOR trial was the first to compare quality of life and survival outcomes between TORS with neck dissection (ND) and primary RT [44]. The primary RT group had a 6.8% improved MD Anderson Dysphagia Inventory (MDADI) score over the TORS with ND group at 1 year post-treatment; however, this 6.8% change was not deemed clinically significant. Both groups had similar oncologic outcomes. Notably, over 70% of the TORS/ND group required adjuvant RT; this population had a marginal 4.3% decrease in MDADI score compared to TORS/ND alone [44,45]. The results of this trial suggest that appropriate treatment modalities for OPSCC should be made on a case-by-case basis [44]. The ORATOR2 trial (NCT 03210103) is currently underway to assess the effect of 2 de-escalation protocols, primary RT (lowered to 60 Gy) versus TORS, on survival and treatment toxicities [46].

#### 2.3.2. Systemic Therapy

Systemic therapy, including chemotherapy and/or immunotherapy, may not be an effective primary definitive treatment modality; rather, it is used in the adjuvant setting concurrent with radiation for high-risk disease as well as for R/M HNSCC. The standard of care chemotherapy for HNSCC is cisplatin, which is a platinum-based alkylating agent [27]. High-dose (100 mg/m^2^) cisplatin given every three weeks may be preferable to low-dose (30 mg/m^2^ every week) regimens, as Noronha et al. [47] demonstrated a superior locoregional control (LRC) rate with the high-dose regimen. In addition to cytotoxic chemotherapies, cetuximab, an anti-Epidermal Growth Factor Receptor (EGFR) monoclonal antibody, was the first targeted agent to gain FDA approval in treating locally advanced HNSCC [48,49]. Cetuximab with platinum-based therapy was later FDA-approved for first-line treatment of R/M HNSCC patients who could not receive RT. This was based off findings from EXTREME, which demonstrated improvement in OS and progression-free survival when cetuximab was added to chemotherapy in R/M HNSCC [48,50]. However, further investigations comparing cetuximab with cisplatin have shown HPV-positive OPSCC patients treated with cetuximab to have inferior overall and progression-free survival than those with cisplatin [51,52]. Furthermore, EGFR inhibition has not proven effective in HNSCC, regardless of HPV status. Siu et al. [53] demonstrated superior overall survival (OS) in patients treated with cisplatin and standard fractionation RT than with panitumumab, an anti-EGFR monoclonal antibody, and accelerated-fractionation RT. Ang et al. [54] also demonstrated that adding cetuximab to a primary radiation-cisplatin regimen in stage III or IV disease did not improve progression-free survival or OS. Therefore, the role of EGFR inhibition in the HNSCC treatment requires further elucidation, and cisplatin remains the standard of care. In the recent years, attention has shifted toward immunotherapy as a new systemic treatment option [55].

Immune-checkpoint inhibitors (ICI), monoclonal antibodies targeting immune checkpoints [programmed cell death protein 1 (PD1), programmed cell death protein 1 ligand (PD-L1), cytotoxic T-lymphocyte-associated protein-4 (CTLA-4)], have changed the treatment landscape of locally advanced and R/M HNSCC. Currently, HNC can be managed by ICIs through pembrolizumab (anti-PD-1) monotherapy if the PD-L1 combined positive score is ≥1%, or in combination with platinum/fluorouracil chemotherapy as a first-line agent for treatment-naïve, R/M HNC. For patients with R/M HNC refractory to platinum chemotherapy, pembrolizumab or nivolumab (anti-PD-1) may be used [27] (Table 2). These indications were based off of results from the KEYNOTE-048 and CHECKMATE-141 trials [56,57]. KEYNOTE-048 demonstrated that a pembrolizumab/chemotherapy combination improved overall survival (OS) over cetuximab/chemotherapy, as well as pembrolizumab monotherapy over cetuximab/chemotherapy [57]. CHECKMATE-141 revealed nivolumab to improve OS and the overall response rate (ORR) in R/M, platinum refractory HNSCC [56].

Multiple biomarkers, including PD-L1 expression, HPV status, and tumor mutational burden have been explored as potential predictors of response to ICI [58]. PD-L1 expression is quantified in several ways, including expression by only tumor cells or only immune cells, or by using a combined positive score defined by the percentage of all tumor and immune cells expressing PD-L1 on immunohistochemistry. Further, various percentage cutoffs from 1% to 20% have been used [58]. In a systematic review including 1088 HNSCC cases, Patel et al. [59] concluded that PD-L1 expression of ≥1% was associated with improved ORR; however, no association was seen based on HPV status. Interestingly, although PD-L1 expression may predict response to ICI therapy, Troiano et al. [60] conducted a meta-analysis and concluded that high PD-L1 expression did not correlate with survival outcomes in OCSCC. Tumor mutational burden, characterized by the number of coding somatic mutations per megabase, has shown promise as both a prognostic feature as well as a predictor to ICI response [58]. In fact, Hanna et al. [61] demonstrated that the tumor mutational burden was significantly associated with improved OS as well as ORR to ICI therapy in HNSCC patients. The utility of all aforementioned biomarkers may improve as methods to characterize them become more standardized.

#### 2.3.3. Treatment De-Intensification in HPV-Positive OPSCC

As HPV-positive patients are generally younger and have a more favorable prognostic outcome than HPV-negative HNSCC patients, attention has been turned toward treatment de-intensification regimens to preserve oncologic outcomes and reduce treatment-related toxicities [37,62]. Multiple trials have de-intensified therapy by reducing both RT and/or chemotherapy dosing in the definitive CRT setting, post-surgical adjuvant setting, or following induction chemotherapy. As distant metastasis remains a predominant pattern of failure for HPV-positive OPSCC, candidacy for treatment de-intensification can include patients at the lowest risk for distant metastasis, patients with a <10 pack-year smoking history, and patients with low T- and N-classification [20,24,63,64]. In fact, An et al. proposed a risk stratification scheme that placed OPSCC into low-, intermediate-, and high-risk categories based on HPV status, T- and N- classification, as well as smoking history [24].

Five trials investigating reduction in RT (to 45–56 Gy) with concurrent chemotherapy based on response to induction chemotherapy yielded similar results in low-risk subgroups, with reduced RT dosing groups showing comparable survival results to regular RT dosing groups [65,66,67,68,69]. In these scenarios, favorable responses to induction chemotherapy may select for increased radio responsiveness and appropriate treatment de-intensification candidates.

In the adjuvant setting, the reduction of RT dosing (to 30–36 Gy) with concurrent docetaxel resulted in comparable LRC and survival, with significant reductions of acute and late toxicities compared to reported standard therapy outcomes [70]. Similarly, sparing of RT to the primary site during adjuvant treatment found reassuring survival (100% OS, 98% 2-year recurrence-free survival [RFS]) and quality of life outcomes [71].

In the definitive CRT setting, two trials also explored the impact of both reduction in RT (to 60 Gy) and cisplatin (to 30 mg/m^2^ weekly) [72,73]. The results of both trials demonstrated favorable survival (95–100% 2–3 year LRC rates) and functional outcomes (median 15 and 10-month gastrotomy tube placement) [72,73]. In the definitive CRT setting, the RTOG 1016 and De-ES-CALaTE HPV trials explored replacing cisplatin with cetuximab to reduce cisplatin-related toxicity [51,52]. As mentioned previously, the cetuximab arms were associated with worse survival and no improvements in treatment-related toxicities [51,52]. The results of these trials affirm the therapeutic benefit of cisplatin over cetuximab and suggest that cetuximab-based de-intensification for HPV-positive OPSCC may not be the optimal approach.

As the aforementioned trials demonstrate a role for de-intensification of RT in various clinical scenarios, future investigation for HPV-positive OPSCC treatment de-intensification is promising. However, these clinical trials lack long-term follow-up data (i.e., ≥5 years) and may obscure the risk of late failures. Multiple ongoing clinical trials, such as the NRG-HN005, are investigating the substitution of cisplatin with immunotherapy [74]. Additional trials are improving risk stratification using pre-treatment (assessing tumor hypoxia burden, NCT03323463 [75]) or mid-treatment (assessing response to therapy) imaging characteristics (NCT03416153, NCT03215719, NCT03224000) [76,77,78]. Further investigation to determine the optimal candidacy for de-intensification as well as the refinement of treatment de-intensification algorithms is warranted.

## 3. Skin Cancer

Cutaneous malignancies of the head and neck are primarily comprised of nonmelanoma skin cancers. These are of epithelial origin and include basal cell carcinoma and cutaneous squamous cell carcinoma. Less commonly encountered, however more aggressive in nature, are cutaneous melanoma and Merkel cell carcinoma. A brief overview of these four skin cancers will be provided here.

### 3.1. Basal Cell Carcinoma

Basal cell carcinoma (BCC) is the most common form of skin cancer, making up 70–80% of cutaneous malignancies [79,80]. BCC often presents at an early stage and has a favorable prognosis with 5-year recurrence-free survival rates of 95% following surgical excision [81]. Surgical modality is based on risk assessment of the BCC lesion, with standard surgical excision reserved for low-risk lesions and Mohs micrographic surgery (MMS) for the excision of lesions in aesthetically and functionally sensitive areas [82]. MMS allows for the retention of cosmesis and function by removing less uninvolved tissue, while wide local excision (WLE) ensures sound oncological resection [83]. For the minority of cases presenting with unresectable/metastatic disease, hedgehog inhibitors (HHIs) as a form of systemic therapy can be used. In fact, there are two FDA-approved HHIs, vismodegib and sonidegib, for unresectable/metastatic BCC [84,85]. Unfortunately, the majority of patients using HHIs have at least one low-grade adverse event such as muscle cramps, alopecia, or dysgeusia [86,87]. Ongoing clinical trials are investigating the efficacy of topical treatment options such as remetinostat, a histone deacetylase inhibitor, (NCT03180528) and of immune checkpoint inhibitors (ICIs) for patients who have progressed on HHIs or other systemic therapies (NCT03132636 and NCT03521830) [88,89,90].

### 3.2. Cutaneous Squamous Cell Carcinoma

Cutaneous squamous cell carcinoma (cSCC) make up 15% of all skin cancers and represent a more aggressive form of nonmelanoma skin cancer than its BCC counterpart [80]. A high-risk subset of cSCC, comprising 5–10% of diagnoses, presents with locally advanced or distant metastatic disease [91]. High-risk pathologic features may include perineural and/or lymphovascular invasion, poor differentiation, desmoplastic or acantholytic histology, and increased depth of invasion. High-risk patient-specific features may include immunocompromised status, location on ear or lip, location in fields of prior radiation treatment, cervical nodal or intraparotid involvement. This list of features is subject to change, as there has been little agreement in defining a high-risk status [91]. Unique to cSCC is the pattern of locoregional spread, as cSCC primaries of the head and neck often spread to intraparotid lymph nodes as well as cervical lymph nodes.

Previous staging systems such as the AJCC7 have not been effective in risk stratification and hazard discrimination of cSCC [91]. The major difficulties with the AJCC7 staging criteria included a lack of prognostic homogeneity within the T2 classification. Additionally, poorer outcomes were often seen in T1/T2 classifications, whereas effective stratification would demonstrate worsening outcomes with the advancing stage of disease [92]. With the AJCC8, T-classification criteria were updated to include important prognostic factors such as tumor thickness >6 mm, perineural invasion ≥0.1 mm, and invasion of cSCC beyond subcutaneous fat [23,91]. The AJCC8 is often compared to an alternative staging system, developed by Brigham and Women’s Hospital, that stratifies T-classification by number of high-risk features [93]. These high-risk features include: tumor diameter ≥2 cm, tumor invasion beyond subcutaneous fat, poorly differentiated histology, perineural invasion of nerves ≥0.1 mm in diameter, and bone invasion (automatically upstaging to T3 classification) [94]. Both the AJCC8 and Brigham Women’s Hospital systems have demonstrated significant utility in risk stratification of cSCC in comparison to the AJCC7 staging system; however, further refinement of staging criteria is warranted. In particular, the aforementioned high-risk features should be further evaluated to determine differing levels of significance.

Standard of care of locally confined, low-risk cSCC is surgical excision with MMS or surgical excision with pathologic margin control without adjuvant therapy. In contrast, the treatment of advanced cSCC is not well standardized. Adjuvant RT is recommended for high-risk features, as described earlier [83,95,96]; however, the addition of concurrent cisplatin to adjuvant RT has not demonstrated an improvement in disease-free nor overall survival [97]. Alternatively, advanced cSCC can be treated systemically with cemiplimab (anti-PD-1 ICI), which was FDA-approved for patients with R/M cSCC in September 2018 (Table 2) [98] based on a trial demonstrating a ORR of 47% [99]. The impact of adjuvant immunotherapy with cemiplimab or pembrolizumab is currently under investigation for patients with high-risk cSCC after surgery and radiation therapy (NCT03833167 and NCT03969004) [100,101].

### 3.3. Cutaneous Melanoma

Cutaneous melanoma represents less than 5% of all skin cancers, yet accounts for up to 60% of skin cancer-related deaths [102]. The AJCC8 introduced multiple changes to the TNM staging criteria of cutaneous melanoma [23]. Tumor thickness must be measured in 0.1 mm increments, as opposed to previous measurements of 10 um increments [23]. T-classification now takes into consideration tumor thickness and the presence or absence of ulceration; notably, the mitotic rate is no longer considered [23]. N-classification is dependent on both clinically occult and clinically detectable lymph node metastasis; these are determined by sentinel lymph node biopsy and physical exam and/or imaging, respectively. Non-nodal regional disease, which includes in transit, satellite, and/or microsatellite metastases, is now incorporated into the N-classification criteria in combination with the numbers of lymph node involved [103]. M-classification is newly subdivided based on the location of distant metastasis, which commonly includes distant skin sites, lung, abdominal organs, and the central nervous system [23,104].

Surgical treatment has been the mainstay for locally confined disease. Unlike in nonmelanoma skin cancer where the role of MMS is well elucidated, there has been an ongoing debate regarding whether MMS or wide local excision (WLE) should serve as the primary surgical modality. MMS may play a role for in situ or less invasive disease, given some reports showing comparable to improved cancer specific and overall survival [105,106]. Future research may be warranted to best select patients appropriate for MMS versus WLE [105,106,107]. Following surgical resection, adjuvant systemic targeted therapy such as ICIs or BRAF/MEK inhibition are warranted and discussed further.

Cutaneous melanoma has a propensity to spread to local lymph node tissues, highlighting the importance of early neck surveillance. The role of sentinel lymph node biopsy (SLNB) in cutaneous melanoma remains ever-changing. Recent updated guidelines from the American Society of Clinical Oncology (ASCO) now offer the option of SLNB for intermediate thickness (1 to 4 mm) tumors. Routine SLNB is not recommended for T1a disease (<0.8 mm without ulceration) [108]. These recommendations were based on reports describing intermediate Breslow thickness as a predictor for SLNB positivity in clinically node-negative (cN0) populations [109,110,111].

**Figure 2:**
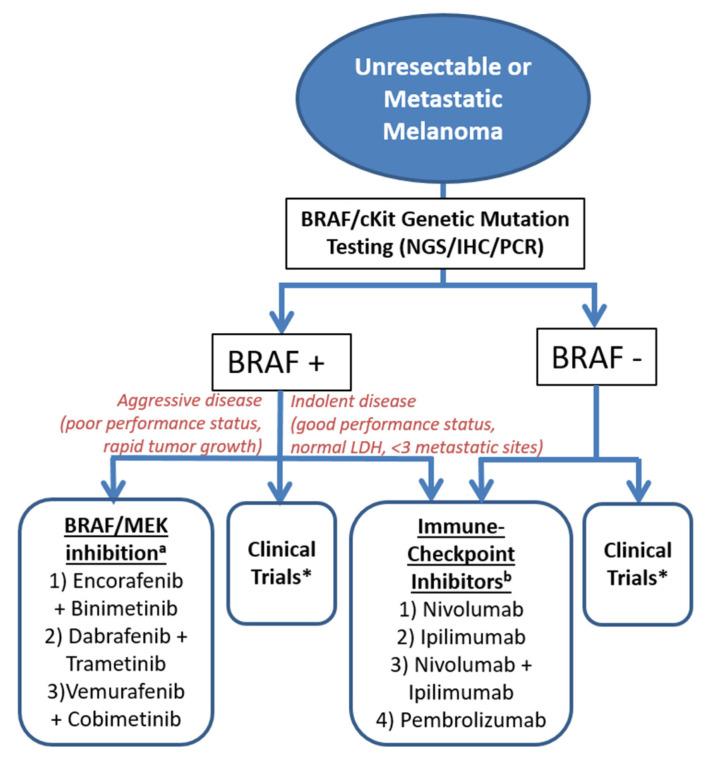
Systemic treatment options for unresectable or metastatic melanoma. **Abbreviations**: BRAF positive for mutation (BRAF+), BRAF negative for mutant type (BRAF−), immunohistochemistry (IHC), lactate dehydrogenase serum level (LDH), mitogen-activated protein kinase (MEK), next-generation sequencing (NGS), polymerase chain reaction (PCR). * Areas under active investigation include, but are not limited to different immunotherapy and BRAF/MEK inhibition regimens, or c-kit targeted therapies. ^a^ FDA-approved for advanced melanoma with BRAF V600E or V600K mutation.

Treatment of advanced melanoma has advanced in recent years, as the role of certain oncogenes (BRAF/NRAS/KIT) in disease pathogenesis has been further elucidated. Patients with metastatic melanoma should undergo testing for the aforementioned genetic mutations to assess candidacy for targeted therapies, as recent mutation-specific therapies have significantly improved treatment responses far beyond the traditional chemotherapy regimens [112]. These therapies have a role both in the adjuvant setting of stage III and also as first-line therapy in metastatic disease [113]. Roughly 50% of melanoma cases express a BRAF mutation (BRAF-MT) and may either be treated with a BRAF/MEK inhibitor combination or ICI therapy (Figure 2) [114,115,116,117,118,119,120,121,122]. Rapidly progressing cases of BRAF-MT advanced melanoma may be better served with BRAF/MEK inhibition [123]. Both BRAF/MEK inhibition and ICIs can be used to treat less aggressive BRAF-MT advanced melanoma; however, ICIs may be favored in this population, as they are generally better tolerated. Individuals progressing on targeted ICI or BRAF/MEK inhibitor therapy with KIT-mutated disease are candidates for imatinib, which is a tyrosine kinase inhibitor [113,124]. Areas currently under investigation include ICI therapy in the adjuvant setting and also ICI therapy in combination with BRAF/MEK inhibition for BRAF-mutant melanoma [125].

### 3.4. Merkel Cell Carcinoma

Merkel cell carcinoma (MCC) is a rare, aggressive, and separate cutaneous neuroendocrine malignancy with a unique AJCC staging system. Studies have shown that one-half of patients will eventually develop locoregional lymph node metastasis and one-third will develop distant metastasis [126,127]. Approximately 60–80% of MCC tumors are associated with the Merkel cell polyomavirus (MCV) infection [128,129,130]. Although not clearly defined, MCV status may play a prognostic role in MCC tumors, highlighting the need for optimal viral detection in order to establish MCV status [131,132]. Options include PCR-based amplification of MCV DNA and IHC staining of a MCV-directed antibody (CM2B4) [131,133]. Future options for post-treatment surveillance may include the tracking titers of anti-MCV Abs, as these levels may correspond with disease recurrence prior to clinically detectable disease [134].

The National Comprehensive Center Network (NCCN) guidelines recommend primary surgical resection of the primary MCC tumor and also an SLNB in all cN0 MCC, as the risk of occult nodal disease even in early stage tumors is high at 25–30% [135]. A patient with a positive SLNB result will require a therapeutic lymph node dissection. On the other hand, a patient with a negative SLNB result will not necessarily need lymph node dissection and can instead be closely observed for regional disease recurrence [135]. The role of adjuvant RT remains unclear given a paucity of prospective randomized control trials; however, adjuvant RT can be considered in the presence of high-risk disease (lymphovascular invasion, immunosuppression, positive margins) or for nodal involvement [135]. Currently, cytotoxic chemotherapies are not routinely used; however, ICIs have proven to be efficacious. Pembrolizumab and avelumab are two FDA-approved options for treating advanced Merkel cell carcinoma (Table 2) [122,136]. Clinical trials are underway investigating the role of ICIs, such as pembrolizumab, in the adjuvant setting (NCT03712605) [137].

## 4. Emerging Developments—Immunotherapy

The treatment of HNC with immunomodulators was not widely accepted early on when compared to other solid-organ malignancies. However, recent developments have seen a number of ICIs FDA-approved across multiple histologies (HNSCC, cSCC, MCC) [98,120,121,122,136]. Table 2 demonstrates an overview of FDA-approved ICIs for HNC. Ongoing trials are investigating the efficacy of ICIs for use in the neoadjuvant, adjuvant, or definitive setting, in combination with other systemic therapies, and through differing modes of delivery (intravenous versus intratumoral injection) [138].

**Figure 3:**
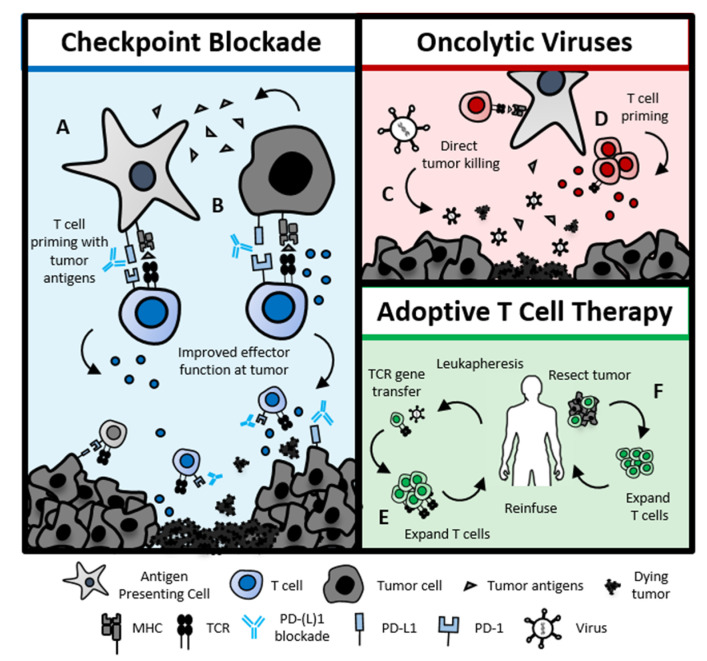
Immunotherapies for head and neck cancers. Checkpoint blockade: (**A**) Programmed cell death protein 1 ligand (PD-1) or PD-L1 blockade has the potential to improve T cell activation after antigen-presenting cells present tumor antigens to T cells or (**B**) may promote T cell effector function directly at the tumor. Oncolytic viruses: Infection of tumor cells with oncolytic viruses promotes tumor death (**C**) by the direct killing of tumor cells, or (**D**) by the release of tumor neoantigens and augmenting immune responses to tumor. Adoptive T cell therapy: Tumors are targeted with a patient’s own T cells, which are either (**E**) expanded directly from the tumor or (**F**) engineered with TCRs responding to tumor antigens. Abbreviations: Major Histocompatibility Complex (MHC); T cell receptor (TCR).

Immunotherapies that further engage both innate and adaptive immunity are also in development (Figure 3). In combination with pembrolizumab, intratumoral injections of oncolytic viruses (talimogene laherparepvec, T-VEC) [139] or Toll-like receptor 9 agonist CpG-ODN [140] have shown promising results and manageable toxicity profiles. Ongoing Phase I/II trials of adoptive T cell therapy, where patients are treated with autologous T cells, have generated responses in some patients. Various T cell products under study include expanded tumor-infiltrating lymphocytes [141] and T cells engineered with T cell receptors targeting E6 and E7 peptides associated with HPV (NCT02280811 and NCT02858310) [142,143].

## 5. Emerging Developments: Radiation Therapy

RT is a mainstay in the multimodality treatment of HNC and is often used in the adjuvant setting for tumors demonstrating high-risk features or, for certain early stage disease, as a primary treatment option for radiosensitive tumors [27]. In advanced, unresectable diseases (recurrence, brain metastasis, or oligometastases [distant metastasis in <5 locations]), reirradiation or stereotactic beam radiation therapy can be considered [144]. Previous traditional 3D conformal therapies have been replaced by intensity-modulated radiotherapy to better delineate target areas while sparing nearby structures [144]. New modalities of RT also include particulate-based (e.g., proton-based) therapy, which may more precisely administer radiation at a targeted depth over standard photon-based therapy [144].

As radiation treatment proceeds, the spatial location, tumor volume, and surrounding tissue may often change. Unfortunately, initial RT planning may not always predict these changes, leading to over/under dosing and dose inhomogeneities [145]. Adaptive radiotherapy involves the re-planning of RT throughout treatment to adjust for changing anatomy. Adaptive radiotherapy can be time-intensive and unrealistic for all patients; however, it can be considered for those with dramatic tumor shrinkage, weight loss, or other obvious anatomical changes [145]. Retrospective studies have shown an improvement in locoregional control with adaptive radiotherapy over standard RT [146,147]. Further investigation is warranted to establish the benefit and feasibility of adaptive radiotherapy.

## 6. Emerging Developments: Cancer Survivorship

Cancer survivorship embodies the long-term care of cancer survivors, including monitoring for disease recurrence/second primaries, the management of acute and long-term side effects, and care coordination between members of the multidisciplinary team [148]. Survivorship is growing in importance given the recent exponential increase in young HNC survivors, secondary to the rising incidence of HPV-related OPSCC [149,150]. Patient-reported outcome measures (PROMs) are instrumental in the survivorship period, as the integration of patient-specific concerns may ensure comprehensive care. PROMs can effectively assess multiple (e.g., overall quality of life, or QoL) or single-domain outcomes [151]. For example, the University of Washington Quality of life (UW-QOL) PROM evaluates domains such as pain, appearance, activity/recreation, speech/swallow/chewing function, and mood/anxiety [152]. Alternatively, the MD Anderson Dysphagia Inventory (MDADI) solely evaluates the impact of swallowing dysfunction on an individual’s daily life [153]. PROMs can alert clinicians to aggravating symptom burdens that otherwise may not have been voiced. In fact, in a study examining the management of metastatic cancer patients, Basch et al. [154] found the integration of PROMs into routine care to be associated with improved symptom management and overall survival.

A cancer diagnosis can cause perspectives to shift, priorities to change, and one’s deepest fears to be realized. Survivors often face physical, emotional, social, and spiritual challenges before and after treatment. With increased psychological distress, HNC patients have a four-time increased risk of death from suicide over the general population and two-time increased risk over non-HNC patients [155,156]. Patients who have undergone major reconstruction are susceptible to body image disorder, manifesting in dissatisfaction in appearance, lowered self-esteem, and tendency toward social isolation [148,157,158]. Active research in survivorship care aims to address these psychosocial effects.

For the first time in history, the American Head & Neck Society’s annual conference, rescheduled to July 2021, will focus on HNC survivorship and is open to any cancer survivor to attend. HNC survivors will be given the unique opportunity to share their experience with other survivors, family members, and healthcare providers, while learning how to best navigate through the survivorship period [159].

## 7. Emerging Developments: Care during the COVID-19 Era

The global COVID-19 pandemic, caused by the novel SARS-CoV-2, has led to over 387,000 deaths worldwide as of June 3rd, 2020 [160]. As a majority of HNCs arise in the mucous membranes of the upper aerodigestive tract, a primary site for SARS-CoV-2 to harbor, the safety of health care providers treating HNC may be jeopardized [161]. Additionally, the effects of COVID-19 on the healthcare system may cause delays in care, from diagnosis to workup to treatment. Since treatment delays for HNC patients have been associated with poor survival outcomes [162,163], effort must be taken to ensure timely treatment. As such, many proposed management algorithms have prioritized the treatment of mucosal as well as advanced staged thyroid and skin cancer [164,165].

To mitigate the risk of infection, healthcare providers should don appropriate personal protective equipment (single-use N95 or PAPR mask, goggles, face shield, gown and gloves) prior to any head and neck examination or procedure [166]. Mady et al. [167] has also described additional preventative strategies to COVID-19 transmission, including the application of povidone–iodine (PVI) through both nasal irritation and oral/oropharyngeal rinses. This is based on evidence showing PVI to inactivate both SARS-CoV and MERSCoV, which are other strains in the coronavirus family [168]. Both patients and healthcare providers can benefit from the application of PVI prior to high-risk procedures or high-risk circumstances for viral transmission. Additionally, surgery may only proceed after the patient has tested negative for SARS-CoV-2 using a polymerase chain reaction (PCR) test [16]. As viral testing may be in short supply, a potential alternative includes serological testing for antibodies developed against SARS-CoV-2; however, a positive result does not necessarily ensure immunity [169]. It is clear that a delicate balance must be struck between providing the best oncologic care while minimizing the risk of COVID-19; recent adaptations by the HNC community show that our field is up to the task [3].

## 8. Conclusions

The landscape of the wide variety of malignancies in the head and neck region is continually changing, from diagnosis to survivorship. Recent developments in staging criteria offer better risk stratification. Advances in treatment modalities are being continually refined to optimize both oncologic as well as quality of life outcomes. As individuals live beyond their cancer, additional work aims to ensure that not just their medical but also psychosocial needs are met. During the COVID-19 era, care for head and neck cancer must proceed in a fastidious fashion to accommodate both oncologic outcomes while accounting for the imminent threat of COVID-19. As the field of head and neck cancer continues to advance, we may afford our patients a better chance at life, one hopefully of the best quality possible.

## Figures and Tables

**Table 1:** Tumor staging of oral cavity squamous cell carcinoma per AJCC8 guidelines.

T-Classification	Tumor Size and Depth of Invasion (DOI)
T1	≤ 2 cm and DOI ≤ 5 mm
T2	≤ 2 cm and 5 < DOI ≤ 10 mm OR >2 and ≤ 4 cm and DOI ≤ 10 mm
T3	>2 and ≤ 4 cm and DOI > 10 mm OR > 4 cm and 0 < DOI ≤ 10mm
T4a	> 4 cm and DOI > 10 mm OR Moderately Advanced Local Disease *
T4b	Very Advanced Local Disease *

Abbreviations: American Joint Committee on Cancer (AJCC); depth of invasion (DOI). * Moderately advanced—invasion of local structures including mandibular/maxillary cortical bone, maxillary sinus, or skin of face; Very advanced—invasion into masticator space, pterygoid plates, or skull base and/or encasement of internal carotid artery.

**Table 2:** FDA-approved immune-checkpoint inhibitors in HNC.

Cancer	Immunotherapy	Indication	Required Diagnostic Testing
HNSCC	Pembrolizumab (Anti-PD-1 Mab)	1st line with FU/platinum-based therapy for treatment naïve R/M	None
		1st line for R/M with PD-L1 [CPS ≥1] expression	PD-L1 (+) Expression with CPS ≥ 1%
		2nd line for R/M after progression on platinum-based therapy	None
	Nivolumab (Anti-PD-1 Mab)	2nd line for R/M after progression on platinum-based therapy	
MCC	Pembrolizumab (Anti-PD-1 Mab)	1st line for R/M	
	Avelumab (Anti-PD-L1 Mab)	1st line for M	
cSCC	Cemiplimab-rwlc (Anti-PD-1 Mab)	1st line for LA/M	
Melanoma	Nivolumab (Anti-PD1 Mab)	1st line for LA/M BRAF-WT and BRAF-MT	
		Adjuvant tx for LN mets/M after primary resection	
	Nivolumab (Anti-PD1 Mab) +Ipilimumab (Anti-CTLA-4 Mab)	1st line for LA/M BRAF-WT or BRAF-MT	
	Ipilimumab (Anti-CTLA-4 Mab)	1st line for LA/M	
		Adjuvant tx for LN mets after primary resection and lymphadenectomy	
	Pembrolizumab (Anti-PD-1 Mab)	1st line for LA/M melanoma	
		Adjuvant tx for LN mets after complete resection	

Abbreviations: BRAF mutant type (BRAF-MT); BRAF wild-type (BRAF-WT); combined positive score (CPS); cutaneous squamous cell carcinoma (cSCC); fluorouracil (FU); head and neck cancer (HNC); head and neck squamous cell carcinoma (HNSCC); locally advanced unresectable disease (LA); Merkel cell carcinoma (MCC); metastatic disease (M); not applicable (N/A); programmed-death receptor 1 (PD-1); programmed-death ligand 1 (PD-L1); recurrent and unresectable disease (R).
